# Utilization of a Voice-Based Virtual Reality Advanced Cardiac Life Support Team Leader Refresher: Prospective Observational Study

**DOI:** 10.2196/17425

**Published:** 2020-03-12

**Authors:** Daniel Katz, Ronak Shah, Elizabeth Kim, Chang Park, Anjan Shah, Adam Levine, Garrett Burnett

**Affiliations:** 1 Department of Anesthesiology, Pain, & Perioperative Medicine Icahn School of Medicine at Mount Sinai New York, NY United States; 2 City University of New York City Medical School New York, NY United States

**Keywords:** video game, experimental game, virtual reality, advanced cardiac life support

## Abstract

**Background:**

The incidence of cardiac arrests per year in the United States continues to increase, yet in-hospital cardiac arrest survival rates significantly vary between hospitals. Current methods of training are expensive, time consuming, and difficult to scale, which necessitates improvements in advanced cardiac life support (ACLS) training. Virtual reality (VR) has been proposed as an alternative or adjunct to high-fidelity simulation (HFS) in several environments. No evaluations to date have explored the ability of a VR program to examine both technical and behavioral skills and demonstrate a cost comparison.

**Objective:**

This study aimed to explore the utility of a voice-based VR ACLS team leader refresher as compared with HFS.

**Methods:**

This prospective observational study performed at an academic institution consisted of 25 postgraduate year 2 residents. Participants were randomized to HFS or VR training and then crossed groups after a 2-week washout. Participants were graded on technical and nontechnical skills. Participants also completed self-assessments about the modules. Proctors were assessed for fatigue and task saturation, and cost analysis based on local economic data was performed.

**Results:**

A total of 23 of 25 participants were included in the scoring analysis. Fewer participants were familiar with VR compared with HFS (9/25, 36% vs 25/25, 100%; *P*<.001). Self-reported satisfaction and utilization scores were similar; however, significantly more participants felt HFS provided better feedback: 99 (IQR 89-100) vs 79 (IQR 71-88); *P*<.001. Technical scores were higher in the HFS group; however, nontechnical scores for decision making and communication were not significantly different between modalities. VR sessions were 21 (IQR 19-24) min shorter than HFS sessions, the National Aeronautics and Space Administration task load index scores for proctors were lower in each category, and VR sessions were estimated to be US $103.68 less expensive in a single-learner, single-session model.

**Conclusions:**

Utilization of a VR-based team leader refresher for ACLS skills is comparable with HFS in several areas, including learner satisfaction. The VR module was more cost-effective and was easier to proctor; however, HFS was better at delivering feedback to participants. Optimal education strategies likely contain elements of both modalities. Further studies are needed to examine the utility of VR-based environments at scale.

## Introduction

### Background

The incidence of cardiac arrests per year in the United States continues to increase, yet in-hospital cardiac arrest survival rates significantly vary between hospitals. Survival rates are reported between 11% to 35%, and patients in hospitals where clinical staff report adequate resuscitation training have greater odds of survival [1]. Health care professionals are often required to have advanced cardiac life support (ACLS) training depending on the institution, but despite training, survival rates are low. Current methods of face-to-face training are expensive, time consuming, and difficult to scale, which necessitates improvements in ACLS training aimed at improving patient survival. The current gold standard for ACLS training involves face-to-face, high-fidelity exercises that allow clinicians to work together to resolve mock resuscitation codes. An instructor observes the group while trainees perform a setlist of tasks for different clinical scenarios. At the end of the session, the team is given feedback on their performance. Although this is the standard for training, there are several limitations to this modality, including the need for lengthy sessions, expensive durable equipment, need for trained personnel, and difficulty with scale.

Virtual reality (VR) has been proposed as an alternative or adjunct to high-fidelity simulation (HFS) in several environments, including engineering [2], sports [3], and aviation [4]. Although it has been studied in the realm of ACLS education, to date, no evaluations have explored the ability of a VR program to examine both technical and behavioral skills and demonstrate a cost comparison [5]. In addition, previous studies involving VR applications in ACLS education relied on additional peripheral devices and did not utilize a fully immersive VR environment [6,7].

### Objectives

Therefore, we set out to examine the utility of a fully immersive VR-based team leader refresher to enhance ACLS skills and compare it with a traditional HFS team leader refresher.

## Methods

### Study Design

**Figure 1:**
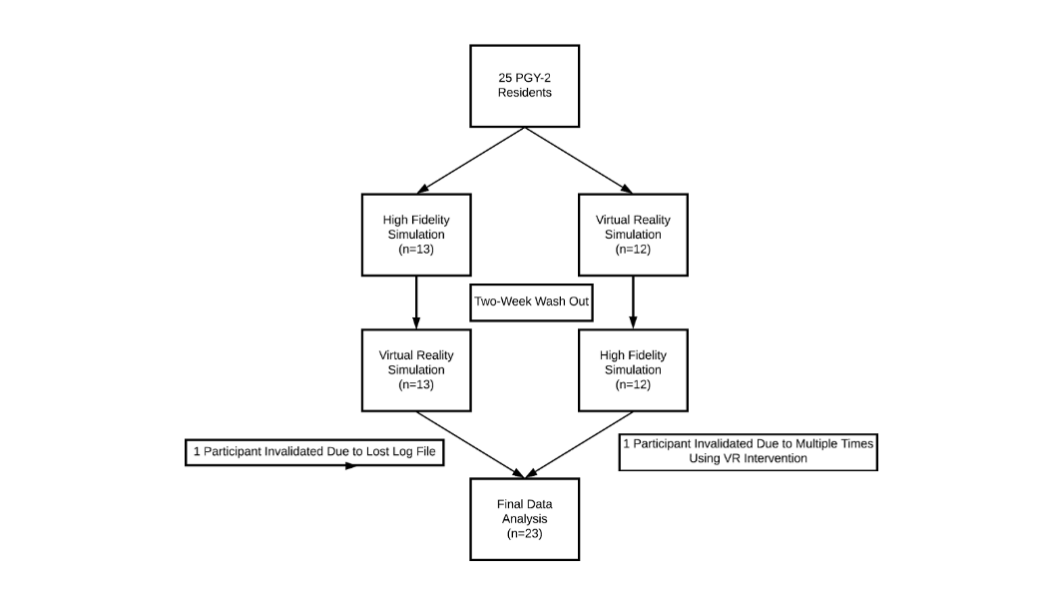
Participant flow diagram.

After obtaining the institutional review board (IRB) approval (IRB 19-02053) and written informed consent, 25 postgraduate year 2 (PGY-2) anesthesiology residents were recruited to participate in our prospective study at the Mount Sinai Human Emulation Education and Evaluation Lab for Patient Safety and Professional Study (HELPS) Center at the Icahn School of Medicine, New York. Each resident was 1 year past their first ACLS certification, had the same clinical rotations the year prior, and had passed the required examinations, and was in good standing with our department. Participants were then randomized into two groups using a balanced random number generator (see Figure 1) based on the first modality utilized. Each group then utilized the alternate modality after a 2-week washout period. A 2-week washout period was chosen to minimize the potential that the residents encountered codes outside of the training environments, yet it still allowed a separation between the two modalities to allow for independent observations of each modality. Study participants and proctors were not told about the purpose of the study. Participants were given no orientation to either modality.

### High-Fidelity Simulation Intervention

HFS sessions were proctored by ACLS-certified instructors who were also board-certified anesthesiologists. All proctors were faculty at the Mount Sinai HELPS Center and had extensive experience with HFS. Instructors were given a rubric of all current American Heart Association (AHA) algorithms to be tested along with a rubric against which to grade in a *Correct, Correct with Assistance, and Incorrect* manner. Algorithms tested included all tachycardia and bradycardia rhythms and algorithms for ventricular tachycardia, ventricular fibrillation, and pulseless electrical activity. Only vocal skills were tested, and only the team leader role was examined. Participants were expected to delegate all manual tasks to other team members, including compressions, airway management, and defibrillation. For example, should the team leader determine compressions were needed, they were expected to tell a team member to begin compressions. After the session concluded, the learner was given a debrief in a structured manner and was given the opportunity to ask any questions. When the debriefing was finished, the session was completed. Simulations were performed on a human patient simulator mannequin (Canadian Aviation Electronics, CAE) using MUSE software (CAE, Montreal, Canada).

### Virtual Reality Intervention

**Figure 2:**
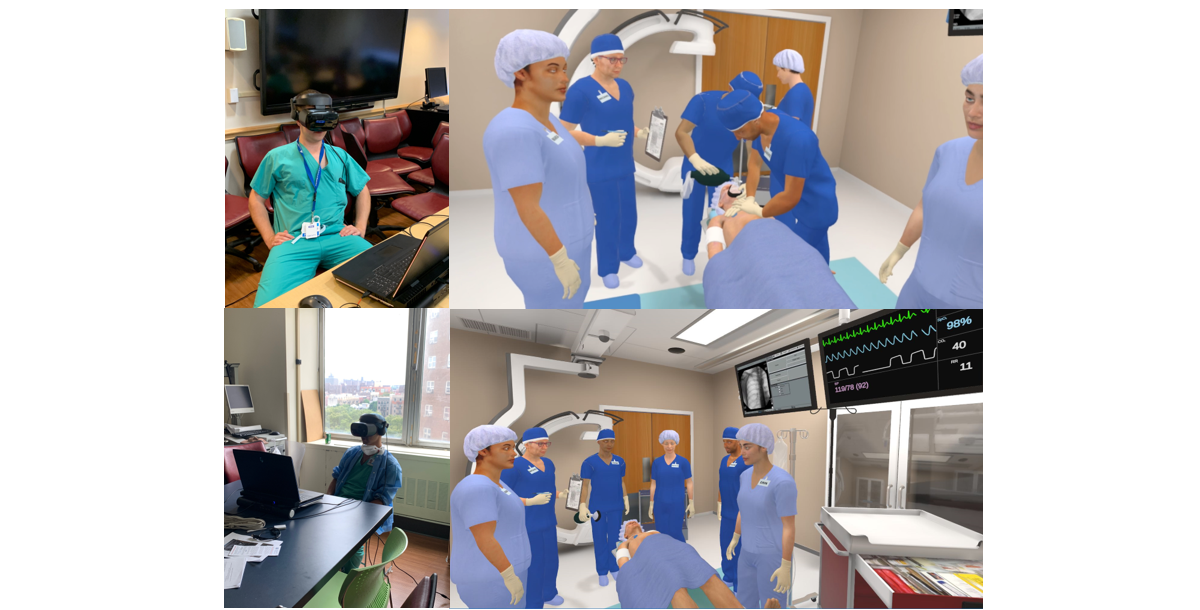
Virtual reality participants and refresher course screen shots.

The VR intervention was an educational module designed by Health Scholars (Denver, CO) that tested participants on the same AHA algorithms as mentioned above. Sessions were run on Dell and HP laptops and Samsung and HP VR headsets using Windows (Microsoft, Redmond, WA) Mixed Reality Software. The session placed the participant in the role of team leader to take care of a critical patient in a radiology suite. The VR intervention utilized voice controls, with a virtual team to which the participant could delegate tasks (Figure 2). Participants were graded on the same rubric as above, including *Correct, Correct with Assistance, and Incorrect*. All of the same scenarios and algorithms were tested as in the HFS group. When the module concluded and the participant removed the headset, the session was completed. As with the high-fidelity arm, only vocal skills were tested, and only the team leader role was examined. Participants were expected to delegate all manual tasks to other team members, including compressions, airway management, and defibrillation. The participants did not interact with the proctor unless there was an issue with the functionality of the system. Participants were given as much time as needed to complete the module. Independent proctors were given transcripts of the VR sessions in the form of log files to determine if incorrect answers were because of a knowledge deficit or because of the voice recognition system misinterpreting the learner.

### Grading Methodology

Each modality was graded against the same rubric (see Multimedia Appendix 1) using the same criteria. Fifteen seconds were allowed to either make a diagnosis or institute a management plan for each item of the algorithm. The only exception to this was the initiating and resuming of chest compressions, which were required to be within 10 seconds. Incorrect answers or answers given after 15 seconds (after which coaching was provided) were graded as incorrect. The scoring mechanism for the VR arm was programmed into the module and exported as a Microsoft Excel file and analyzed. Voice capture output by the natural language processing (NLP) was manually double-checked for every wrong answer to ensure that an incorrect score was not given because of the failure of the NLP to translate speech. The proctor for the HFS performed the grading for the simulations using the same rubric mentioned above. For nontechnical skills (NTSs), proctors in the simulation were given a behaviorally anchored rating scale (BARS) and a sheet containing examples and expected behaviors for each scoring domain. In the VR intervention, BARS outputs were determined by analyzing the voice outputs and placing them into categories along the scale. For example, the score in communication was determined by the percentage of the time that the participant used team member names when communicating. Scores for each domain were tallied and analyzed. Self-assessment scores and feedback on the comparator arms were obtained immediately after the debrief portion of the exercise through survey administration. Our primary outcome was set to be technical skills scores, as measured by the correct percentage of items within 15 seconds without coaching.

### Proctor Assessment

At the end of sessions each day, proctors were asked to fill out a National Aeronautics and Space Administration task load index (NASA-TLX) form indicating their performance and experience throughout the day. The NASA-TLX is a validated instrument for measuring perceived workloads for performing tasks that are graded on a 20-point scale in six domains [8]. Scores were analyzed in each domain. Proctors were allowed to take breaks as needed, including a 45-min lunch break each day. The time required to complete each group was also notated.

### Cost Analysis Methodology

Cost data were obtained from purchasing orders for equipment and were based on predicted salaries for personnel involved. Salaries were adjusted based on the minimum certifications and expertise needed to perform the task. For example, even though the ALCS instructor was a board-certified anesthesiologist, the calculated salary line was adjusted to be in line with ACLS instructors in our area (New York Metro Area). Certain items such as insurance and building costs were not included in the assessment.

### Statistical Methods

Normally distributed variables are presented as mean (SD) with nonnormally distributed variables reported as median (IQR). Normality testing was performed via Shapiro Wilk testing and visual inspection of histograms. Appropriate statistical tests were performed based on normality and qualifying for assumptions. All tests were performed with SPSS version 24 (IBM).

## Results

### Participant Results

Of the 25 participants recruited, 23 were included for the final analysis. One participant erroneously went through the VR intervention multiple times in one sitting, and another participant’s VR log file was lost during the study (see Figure 1). All study participants were aged between 25 and 35 years, and 68% (17/25) of them were male (see Multimedia Appendix 1). There was no difference in baseline comfort or experience with leading or participating in codes (see Table 1). It should be noted that after 1 year of training, no participant had experience in leading a code. Significantly more participants had prior simulation experience (25/25, 100%) compared with those with prior VR experience (9/25, 36%; *P*<.001, Fischer exact test). There was no difference between groups with regard to reality or enjoyment of the experience. Similarly, there was no difference in preference for using either modality to train, and the willingness to use either modality every 6 months was high: 100% (25/25) HFS vs 96% (24/25) VR; *P*=.72 (Fischer exact test; see Table 2). Significantly more participants rated the HFS debrief as providing better feedback (HFS: median 99.0, IQR 89.0-100.0 vs VR: median 79.0, IQR 71.0-88.0; *P*<.001), and there was a nonsignificant trend toward participants rating HFS as more useful in teaching ACLS skills (HFS: median 90.0, IQR 83.0-99.5 vs VR: median 83.0, IQR 80.0-90.5; *P*=.080). Our primary outcome, as measured by scores in technical domains, as measured in percentage correct without assistance, was significantly lower in the VR group than in the HFS group (HFS: median 72.7, IQR 60.0-78.2 vs VR: median 47.0, IQR 40.0-58.0; *P*<.001; Mann-Whitney U Test). Scores were not dependent on the first modality encountered (VR first: median 40.5, IQR 35.5-42.75 vs HFS first: median 38.0, IQR 32.0-44.5; *P*=.810). In nontechnical domains, scores in decision making and communication were no different; however, situational awareness scores were rated lower in the VR group (see Table 2). The overall accuracy of the voice recognition system was very good, with less than 2% of scoring modifications based on incorrect interpretations.

**Table 1:** Baseline information of prior experiences.

Variable	HFS^a^ first (n=13)	VR^b^ first (n=12)	*P* value
I feel comfortable running a code (out of 100), median (IQR)	12 (0-21.5)	18.5 (10.25-26.5)	.27
How many codes have you run, median (IQR)	0 (0-0)	0 (0-0)	.54
How many codes have you participated in, median (IQR)	6 (5-10)	9 (5-15)	.32
Have you participated in HFS? (Yes), n (%)	13 (100)	12 (100)	>.99
Have you used VR before? (Yes), n (%)	4 (31)	5 (42)	.57

^a^HFS: high-fidelity simulation.

^b^VR: virtual reality.

**Table 2:** Self-reported results and session scores.

Variable			High-fidelity simulation (n=25)	Virtual reality (n=25)	*P* value
How real was the experience, median (IQR)			62.0 (50.5-70.0)	50.0 (44.5-66.0)	.13
How useful was the experience in teaching you how to run a code, median (IQR)			90.0 (83.0-99.5)	83.0 (80.0-90.5)	.08
How useful was the feedback received, median (IQR)			99.0 (89.0-100.0)	79.0 (71.0-88.0)	<.001
I enjoyed the experience (Yes), n (%)			23 (92)	22 (88)	.63
I would like to use this as a way to recertify my ACLS^a^ (Yes), n (%)			25 (100)	23 (92)	.14
Was this experience as valuable as your live training for Mega Code the last time you had to recertify? (Yes), n (%)			25 (100)	22 (88)	.25
I would do this once every 6 months to refresh my skills if it was NOT required but I was given time to do so (Yes), n (%)			25 (100)	24 (96)	.72
**Scored domains^b^**					
	Total correct percentage technical domains, median (IQR)		72.7 (60.0-78.2)	47.0 (40.0-58.0)	<.001
	**Nontechnical domains, median (IQR)**				
		Situational awareness	6.0 (5.0-7.0)	1.0 (1.0-1.0)	<.001
		Decision making	6.0 (4.0-6.0)	6.0 (4.0-6.0)	.52
		Communication	5.0 (4.0-6.0)	4.0 (1.0-6.0)	.09

^a^ACLS: advanced cardiac life support.

^b^For scored domains, n=23 each for high-fidelity simulation and virtual reality.

### Instructor Results

The instructor task load was significantly higher for the HFS proctors in every domain tested (see Table 3) in the NASA-TLX. On average, VR sessions were 21 (IQR 19-24) min shorter than the HFS sessions and were US $103.68 less expensive. Table 4 demonstrates the estimated cost difference depending on the number of learners and sessions for a variety of theoretical institutions. Including the time taken to logistically organize the participants to come to the simulation laboratory, each VR group required less than 1 day to complete. Each simulation group required 2 or 3 working days to complete the exercise.

**Table 3:** National Aeronautics and Space Administration task load index data.

Variable	High-fidelity simulation proctors (n=20), median (IQR)	Virtual reality proctors (n=5), median (IQR)	*P* value
Mental demand	12.5 (9.6-15.0)	2 (2-2.5)	<.001
Physical demand	10.0 (5.0-13.7)	3.0 (2.0-3.5)	<.001
Temporal demand	14 (10.2-16.0)	3.0 (2.0-4.0)	<.001
Performance impact	8.0 (6.2-12.0)	2.0 (1.0-3.0)	<.001
Effort	13.0 (12.2-15.0)	2.0 (2.0-3.0)	<.001
Frustration	14.0 (6.7-16.0)	2.0 (2.0-2.5)	<.001

**Table 4:** Time and cost analysis.

Variable	High-fidelity simulation group	Virtual reality group	Difference^a^	Percentage difference
Time per session (min), median (IQR)	42 (38-44)	20 (18-21)	21 (19-24)	50
Cost for single learner, single session (US $)^b^	193.00	89.32	103.68	54
Cost for 50 learners, single session (US $)^b^	9650.00	4466.15	5183.85	54
Cost of 1000 learners, single session (US $)^b^	193,000.00	89,322.92	103,677.08	54
Cost for single learner, four sessions (US $)^b^	772.00	132.29	639.71	83
Cost for 50 learners, four sessions (US $)^b^	38,600.00	6614.58	31,985.42	83
Cost for 1000 learners, four sessions (US $)^b^	772,000.00	132,291.67	639,708.33	83

^a^Median difference in time calculated via Hodges-Lehman median difference.

^b^Cost estimates are based on purchase orders for equipment and salaries for New York Metro Area and are in US $.

## Discussion

### Technical and Nontechnical Skills

The AHA scientific statement by Cheng et al [9] demonstrated that the current strategy for teaching and maintaining ACLS skills must change. It is clear that the frequency of refresher training is inadequate to maintain skills and that our current teaching modalities may have negative impacts on survival [9]. Our study supports previous studies demonstrating that learners believe that HFS is a great teaching modality for ACLS skills [10]. Despite this, HFS as a refresher modality is prohibitively expensive, time consuming, and places a large burden on instructors. This is where new technologies such as VR can be implemented. Our study is not the first to explore VR as a means of teaching ACLS skills, but it does provide new insight into the topic.

Studies by Khanal et al [5], Khanal and Kahol [11], Creutzfeldt et al [12,13], and Semeraro et al [6] have demonstrated the effectiveness of virtual environments to train ACLS skills; however, our study differs in the study subjects and methodology. The largest study was performed by Khanal et al [5] and included 148 participants. Their team demonstrated that VR could lead to enhanced performance in simulated scenarios graded by ACLS experts at varying levels of user feedback. The level of experience of the subjects was not disclosed, and the intervention was a team-based VR experience with peripheral equipment such as joysticks. Our study included PGY-2 residents and was a standalone VR experience. It did not require a team to play, nor did it require controls or joysticks that would enhance the utilization and uptake and make scaling easier. The second study by Khanal and Kahol [11] had 11 participants and was a mixed reality environment (VR and a haptic device). Their team demonstrated the effectiveness of the experience; however, its generalizability is limited by the need of a haptic device. The studies by Creutzfeldt et al [12,13] and Semeraro et al [6] again demonstrated proof of concept; however, their VR experiences required the use of peripheral devices or mannequins in conjunction with VR.

Our study is the first to compare a fully immersive, stand-alone, voice-controlled experience to HFS, and it has demonstrated important findings. Our primary outcome, technical scores for the algorithms, were lower in VR than those in HFS. The source of this difference is likely multifactorial. First, this may have been an artifact because of a lack of familiarity with the environment. All of our residents have experience and comfort with HFS; however, the minority had experience with VR. Furthermore, we did not design or mandate an orientation to the VR module. This unfamiliarity may have contributed to the lower scores. In addition, although both systems used the same rubric for scoring, it may be that in the VR, grading was more stringent. A human grader might interpret *almost* correct responses as correct. Further, human graders may be giving subtle feedback to learners within the grading timeframe. This could be demonstrated by body language cues, a change in voice pitch or timing, or through some other mechanism. Moreover, the inability of the VR system to recognize subjects’ vocal responses could potentially have limited scoring when compared with HFS; however, on manual recheck, this impact was minimal, and as such, this was not likely contributory. VR also offers a level of granularity of assessment in real time that would be impossible for a human proctor to detect, especially when also driving the experience. This might result in more stringent grading, as mentioned above. For example, the VR system can note the difference between taking 30 vs 31 seconds to respond and grade accordingly, whereas a human proctor could not. We opted not to use a third party to grade the HFS group for this reason, as it would be impractical and not congruent with current practice and would have further inflated the cost of the HFS group. Further works are needed to elucidate the differences between human and computerized grading schema for technical domains.

In terms of baseline comparisons, it was interesting but not surprising that none of the participants had code leader experience after 1 year in practice. Second, although all participants in the study had experience with HFS, only 36% (9/25) of participants had experience with VR. There was no orientation to VR for the study, which may have put the VR experience at a disadvantage and may have partially explained the reason for the decrease in scoring, as discussed above. Satisfaction with both experiences was very high, but there was a clear advantage to HFS in the feedback domain. This is not surprising because the HFS group had a full formal debrief, whereas the VR group received only the feedback on items missed in a binary manner. The less effective debriefing could have an impact on knowledge retention. Our study was not designed to evaluate retention; however, it would be important to examine this in future studies. In this regard, VR lags behind HFS and more work needs to be done to enhance the feedback given to learners. Experienced debriefers can tailor the feedback type and technique to individual learners, whereas the VR experience can only deliver feedback one way in its current form. Although this is a clear limitation of VR in this module, there are ways in which this can be improved in the future with better software. Despite this limitation, participants believed the VR experience was as valuable as their live training for recertification. More importantly, the participants were very willing (24/25, 96%) to use VR as a refresher even if it was not required, despite the aforementioned disadvantages.

There were similarities in NTSs as well, which are an important component of acute care and have been shown to impact outcomes in other areas [14]. Our VR experience was able to grade participants in three nontechnical domains [15]. When compared with HFS scores, both decision making and communication scores were not different. This is one of the first indicators that we can use virtual environments to identify and stratify participants by NTSs. However, not every domain correlated, such as situational awareness. On the basis of our study design, we cannot know which of these assessments (HFS or VR) is most correct for this domain; similarly, we can only hypothesize why the situational awareness scores were much lower in the VR group than in the HFS group. It is our belief that this is most likely because of the mechanism by which the VR experience is graded on this domain and not that a VR-based experience is unable to grade a domain such as situational awareness.

### Instructor Fatigue and Cost

Our analysis also demonstrated what is known anecdotally about trying to scale HFS to accommodate the need for frequent refreshers. As demonstrated, proctoring HFS is demanding on proctors, as evidenced by high NASA-TLX scores in the HFS group. It would be difficult to sustain a model of multiple refreshers in a year without causing burnout and fatigue of the staff. Proctors for the VR sessions experienced minimal fatigue. Furthermore, although all proctors were ACLS instructors, proctors for future VR sessions would only need training in using the VR system and not require a mastery of the content. The use of a VR proctor with minimal training would further decrease the cost associated with ACLS refresher courses.

Our time and cost analysis demonstrated that VR sessions can accomplish learning objectives in a shorter time than an HFS session. An advantage like this could be extremely important, as a VR session could be completed during a short coffee break, whereas an HFS session would take twice as long and require practitioners to give up their lunch breaks or come in on off hours to train. Finally, as the frequency of refresher training increases or the number of learners per session increases, the cost savings amplify. One proctor could supervise multiple VR sessions; however, the converse is not true for HFS.

### Advantages of Each Modality

On the basis of our analysis, there are apparent advantages of HFS and VR: HFS provides very high-quality education but at high costs and low scale, and VR-based education currently may lag behind HFS but is more cost-effective and more easily scaled. It is the opinion of the authors that the interpretation of our findings is that each modality has its strengths and weakness, and neither of them is a panacea. As it currently stands, we would recommend that practitioners could utilize a VR medium as a means of a refresher or to screen for those who need more in-depth retraining. If during the VR module, it is discovered that the learner needs more in-depth remediation, one could then deploy the more expensive HFS alternative. In this manner, an institution can provide quality education at scale, while allowing for more targeted programs for those that need extra attention. Another option would be to develop some methodology for reviewing how a participant performed utilizing screen sharing technology; however, this would increase the cost, and it may be more effective simply to hold another session. Finally, as technology improves, it may be possible to provide HFS levels of directed feedback to learners and close this gap. Further development and research are needed before a conclusion should be drawn.

### Limitations

Our study has several limitations. First, as the number of subjects is relatively low, the findings should be interpreted with caution. As stated, our study is exploratory in nature. Second, we utilized a specific VR experience (ACLS VR) by one company (Health Scholars). Other VR experiences for ACLS exist, but our findings may be specific to this experience, which may limit their generalizability. Our participants were all PGY-2 residents at a major academic center. Although we do not believe that our findings would not translate to other practitioners, we cannot say for certain. Finally, a major limitation of our work is that this VR experience does not include testing for hands-on skills such as the ability to perform chest compressions. Although this was not the goal of this experience, it would not be able to replace current training methodologies unless it was paired with a part-task trainer capable of grading these techniques.

### Conclusions

Our study highlights some of the differences and similarities between VR and HFS for team leader refresher training. Scores were lower in the VR module, although the implications of this are unknown. NTSs were similar in some domains but different in others. The VR module was more cost-effective and was easier to proctor; however, HFS was better at delivering feedback to participants. Both modalities demonstrated high levels of satisfaction and a similar willingness for participants to use each modality. Further studies are needed to examine the utility of VR-based environments at scale.

## Appendix

Multimedia Appendix 1 ACLS Scoring Matrix.

## Abbreviations

ACLS: advanced cardiac life support
AHA: American Heart Association
BARS: behaviorally anchored rating scale
CAE: Canadian Aviation Electronics
HELPS: Human Emulation Education and Evaluation Lab for Patient Safety and Professional Study
HFS: high-fidelity simulation
IRB: institutional review board
NASA-TLX: National Aeronautics and Space Administration task load index
NLP: natural language processing
NTS: nontechnical skill
PGY: postgraduate year
VR: virtual reality
