# Supplementary figures and images for: Utilization of a Voice-Based Virtual Reality Advanced Cardiac Life Support Team Leader Refresher: Prospective Observational Study
Source: J Med Internet Res. 2020 Mar 12;22(3):e17425. doi: 10.2196/17425 (PMC7099400; doi:10.2196/17425)

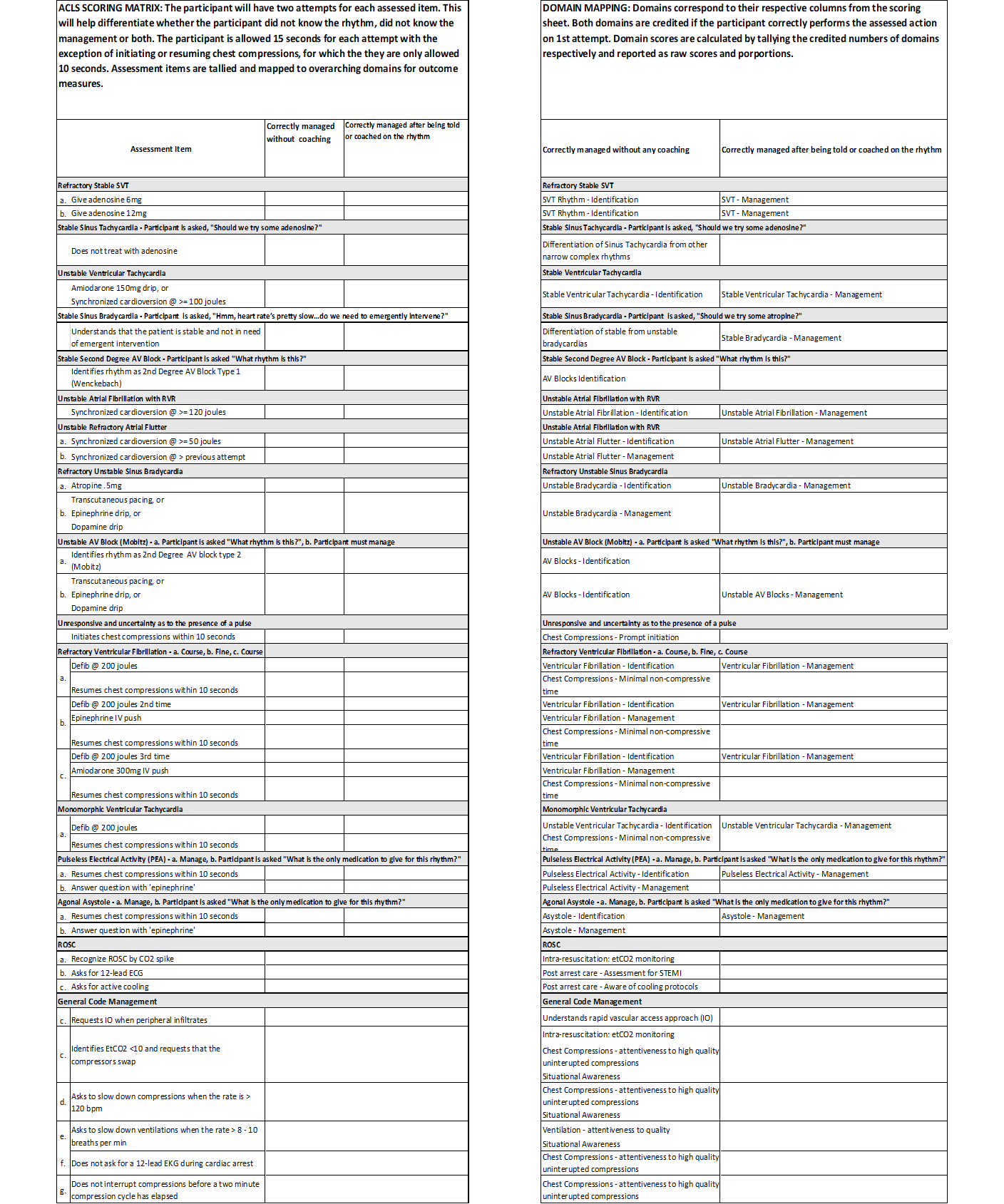

Supplement: Multimedia Appendix 1 [file jmir_v22i3e17425_app1.png]
